# Postural effects of symmetrical and asymmetrical loads on the spines of schoolchildren

**DOI:** 10.1186/1748-7161-2-8

**Published:** 2007-07-09

**Authors:** Stefano Negrini, Alberto Negrini

**Affiliations:** 1 ISICO (Italian Scientific Spine Institute), Via Carlo Crivelli 20, 20122 Milan, Italy; 2 Don Carlo Gnocchi Foundation, ONLUS, Care & Research Institute, Via Capecelatro 66, 20100 Milan, Italy

## Abstract

The school backpack constitutes a daily load for schoolchildren: we set out to analyse the postural effects of this load, considering trunk rotation, shoulder asymmetry, thoracic kyphosis, lumbar lordosis, and sagittal and frontal decompensation from the plumbline. A group of 43 subjects (mean age = 12.5 ± 0.5 years) were considered: average backpack loads and average time spent getting to/from home/school (7 min) had been determined in a previous study conducted on this population. Children were evaluated by means of an optoelectronic device in different conditions corresponding to their usual everyday school backpack activities: without load; bearing 12 (week maximum) and 8 (week average) kg symmetrical loads; bearing an 8 kg asymmetrical load; after fatigue due to backpack carrying (a 7-minute treadmill walking session bearing an 8 kg symmetrical load). Both types of load induce changes in posture: the symmetrical one in the sagittal plane, without statistical significant differences between 8 and 12 kg, and the asymmetrical one in all anatomical planes. Usual fatigue accentuates sagittal effects, but recovery of all parameters (except lumbar lordosis) follows removal of the load. The backpack load effect on schoolchildren posture should be more carefully evaluated in the future, even if we must bear in mind that laws protect workers to carry heavy loads but not children, and results in the literature support the hypothesis that back pain in youngsters is correlated with back pain in adulthood

## Background

Non specific low back pain in schoolchildren is a topic of growing prominence in the literature [1-5]. The scientific community has recently been alerted to the problem of backpacks (a characteristic daily load for subjects in this age group), both because they proportionally exceed legal limits set for adult workers [6-9] and because they seem to be related, although not directly, to low back pain [7]. The problem if backpacks can generate or not back pain is controversial, and today there is a lack of proves either ways [1,2,4,10]. Postural variations have been considered as possible risk factors for low back pain in schoolchildren, even though their relationship to hyperlordotic posture is uncertain in some studies [11-13], with hyperkyphotic posture appearing not to constitute a risk factor [11-13]. Loading – particularly non axial loading – of the spine prompts postural adjustments [14,15] but only recently a few studies have considered the biomechanical implications of backpack carrying in populations of schoolchildren. Pascoe et al. [14] studied a population of 11–13 year olds, demonstrating, through a three-dimensional approach, how a backpack alters, significantly, posture and gait. Grimmer et al. [15] analysed modifications of the head-neck angle, while Hong and Brueggeman [16] considered the problem solely from the gait analysis perspective. Chow recently developed a series of studies on the topic, focusing on load carriage both in normals and adolescent idiopathic scoliosis patients [17-21].

When considering the possible relationship between non specific low back pain, posture and backpack carrying in a population of schoolchildren, it is also necessary to evaluate postural adjustments due to the (symmetrical and asymmetrical) loading of their spines and to the fatigue caused by backpack carrying. The aim of the present study was therefore to reproduce the 'loading conditions' seen in schoolchildren daily and to investigate the instantaneous postural adjustments they prompt.

## Methods

### Subjects

We considered a group of 43 subjects (18 females, 25 males) with the following characteristics: age 12.5 ± 0.5 years; weight 50.2 ± 12.3 kg; height 153.9 ± 8.5 cm; BMI 21 ± 3.8 kg/m^2^. All the children underwent a medical evaluation and were found to be normal, that means without detectable sign or symptoms, actual or previous, that could lead to the suspect of any pathological orthopaedic, neuro-muscular, pneumological and cardio-vascular condition. We also evaluated the average and average maximal (per week) weights of their school backpacks: 9.06 kg (range 4.41–12.29) and 11.33 kg (range 6.77–14.6) respectively; the average of the week load was 21.8% of the body weight, while the average of the weekly maximum weight carried was 27.3% [6].

### Acquisition device

**Figure 1:**
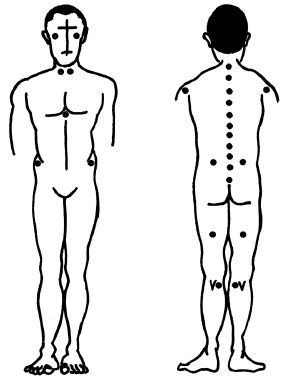
Markers placement on anatomical landmarks of interest.

Data were collected using the **Au**tomatic **Sc**oliosis **An**alyser (**AUSCAN) **System [22], an optoelectronic device, which performs, at a sampling rate of 100 Hz, real-time detection of passive retroreflective markers positioned on the anatomical landmarks of interest (Figure 1). The system computes the three-dimensional coordinates of these markers, and its errors have been already calculated in previous studies and shown to be very low [23-25]. Being optoelectronic, the AUSCAN System is non invasive (non ionising) and does not interfere with physiological parameters.

### Protocol

On the basis of previous evaluations [6], loads of 8 (correspondent to 16.8 ± 3,8% of body weight in this sample) and 12 kg (25.2 ± 5.7% of body weight) were taken because they represent the average and maximal loads carried on a week basis by these schoolchildren. We considered two loading conditions: using two straps (8 kg SYM and 12 kg SYM) and one strap (8 kg ASYM, the shoulder used was chosen by the subjects). All the subjects were evaluated in the upright position, both barefoot and wearing their usual sports shoes: we did not require any particular arm position and they were allowed to chose what they preferred. The data acquisition protocol was made up of three parts, as set out in Table 1. We did not randomised the sequence of measurements, that followed what reported in Table 1, to avoid having the most fatiguing situations (mainly treadmill, but in some respects also asymmetrical load) at the beginning; we tried to avoid the carry-over (as well as a possible training) effects giving pauses between the acquisitions, that lasted in any case a few seconds: anyway we must bear in mind that these weight and times were usual for these children. The marker placement reliability had been previously studied [23], and was performed by a very well trained examiner with years of expertise.

**Table 1:** Data acquisition protocol. SYM: Symmetrical load; ASYM: asymmetrical load. Pauses were given between the different acquisitions to avoid carry-over effect

PART I, pre fatigue	PART II, fatigue	PART III, post fatigue
without load, barefoot, 1 second	(not acquired) 7 min walking on a treadmill at self-selected speed bearing 8 kg load SYM	8 kg SYM, wearing shoes, 1 second
without load, barefoot, 20 seconds		without load, wearing shoes, 1 second
8 kg SYM, barefoot, 1 second		without load, barefoot,1 second
without load, wearing shoes, 1 second		without load, barefoot, 20 seconds
8 kg ASYM, wearing shoes, 1 second		
12 kg SYM, wearing shoes, 1 second		
8 kg SYM, wearing shoes, 1 second		

The duration of the treadmill session (or 'walking time') was 7 min, which corresponded to the average time taken by the children to get to or from school (a mean value established on the basis of a back pain prevalence and backpack management questionnaire previously administered to these subjects) [6-8]. No AUSCAN System data were acquired during the 'walking time'.

**Figure 2:**
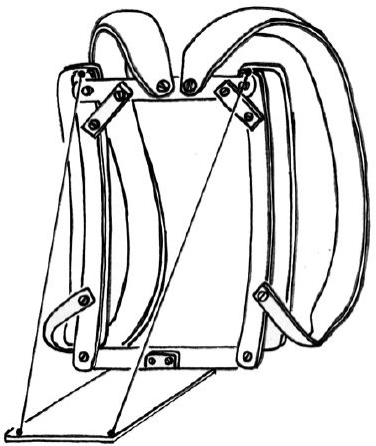
The 'backpack simulator': the aluminium vertical bars were rounded so to follow the trunk profile and avoid incorrect distances from the trunk.

The complete evaluation took about 30 min. In order to preserve the visibility of the markers placed on the spine, a 'backpack simulator' (two 4 – or 6 -kg steel blocks fixed to a frame, see figure 2) had to be used to reproduce the symmetrical loading condition, while a normal sports bag was used to reproduce the asymmetrical loading condition.

### Parameters evaluated

The parameters of interest extracted are defined in Table 2, which also reports units and sign conventions. For the purposes of the present study, to better simulate real life conditions, all parameters were extracted from the "1 second, wearing shoes" data acquisitions in the different conditions, and only the markers positioned on the spinous processes of every other vertebra (from C7 to S3), on the acromia, and on the Posterior Superior and Anterior Superior Iliac Spines were considered. The repeatability of the AUSCAN System in measuring the parameters evaluated in the study have been shown "in vivo" (due to postural variations) to be 6 and 12 mm for plumbline in the frontal and sagittal plane respectively, and 6 mm for shoulders height [24]; the repeatability for trunk rotation have been measured as 3° [26], while for the Cobb angles they ranged between 5 and 7° [23,25].

**Table 2:** Parameters considered. Plumbline = distance of C7 from the vertical plumbline tangential to S1. ASIS: Anterior Superior Iliac Spines.

**Sagittal plane**	**Frontal plane**	**Horizontal plane**
Plumbline (mm). Positive value: C7 forward	Plumbline (mm). Positive value: C7 to the right	Angle of trunk rotation (deg). Positive value: right shoulder forward (ref. ASIS line)
Angle of thoracic kyphosis (deg). Positive value: increase of kyphosis	Asymmetry of shoulders (mm). Positive value: left shoulder up. (ref. ASIS line)	
Angle of lumbar lordosis (deg). Positive value: increase of kyphosis		

### Data analysis

We performed paired data statistical analyses. To evaluate only load effects, we considered data from PART I (Table 1) of the protocol, subtracting basal (without load) values from the loaded condition values; to evaluate the effects of fatigue we considered the differences between corresponding acquisitions in PARTS III and I of the protocol (i.e.,'8 kg SYM', post vs pre). All the data in the tables are presented as mean values ± standard deviations. For a statistical reason, the variance analysis for repeated measurements required to eliminate all students in which it was not possible, for one reason or another (mainly backpack simulator interference), to calculate all needed parameters for the analysis considered: this drove to a reduction of population according to the different situations. Moreover, we did not perform any sub-group analysis (such as gender effect) due to the reduction of numbers that did not granted enough statistical power. The statistical analysis (α = 5%) was performed using the following tests: Shapiro-Wilk's in order to asses the distribution of the data, and, according to this, ANOVA for repeated measures, Friedman rank test, Newman-Keuls post hoc, t-test for paired data and Wilcoxon rank test. The software used was: Matlab 5.1, Excel 7.0, and Statgraphics 3.0.

## Results

We observed that the postural response to the asymmetrical load (8 kg ASYM) was a retropositioning (4 mm) and an elevation (2.5 cm) of the loaded shoulder together with a lateral deviation of the trunk (3 cm) away from the load (Table 3). The results were the same regardless of which shoulder was used (right or left). No statistical significance was reached in the 'left shoulder' subgroup because of the small number of subjects it contained.

**Table 3:** Changes from the neutral position during asymmetrical load: postural effects in horizontal and frontal planes. The results are presented according to the sign convention used in table 2.

	**Right (no. = 27)**	**Left (no. = 4)**
**Trunk rotation**	-4.4 ± 5.1 *	4.3 ± 1.8 *
**Shoulder asymmetry**	-24.6 ± 17.5 *	24.5 ± 28.6
**Frontal plumbline**	-30.6 ± 14.4 *	39.2 ± 41.2

*, Statistically significant difference vs neutral position.

In the 8 kg SYM loading condition, we observed a forward inclination of the trunk (increase of the distance from the plumbline at C7), and a reduction of the angles of lumbar lordosis and of kyphosis, the latter not significant (Table 4). No statistically significant variation in the postural response was found increasing the SYM load from 8 to 12 kg: the 12 kg load produced a slight increase in all the parameter values in the sagittal plane and a lateral trunk inclination (increase of the distance from the plumbline at C7) of 4 mm.

**Table 4:** Changes from the neutral position during different loading modalities: postural effects in the three planes.

	**12 kg SYM**	**8 kg SYM**	**8 kg ASYM**
**Trunk rotation (no. = 30)**	0.01 ± 2.02 †	0.003 ± 1.76 †	3.1 ± 3.4 *
**Shoulder asymmetry (no. = 30)**	-1.2 ± 7 †	-0.03 ± 7.2 †	18 ± 17.8 *
**Frontal plumbline (no. = 30)**	4.3 ± 7.7 *†	1.9 ± 6.5 †	28.3 ± 16.7 *
**Sagittal plumbline (no. = 24)**	76.9 ± 38.1 *†	73.8 ± 27.9 *†	21.9 ± 18.1 *
**Angle of lumbar lordosis (no. = 24)**	-16.3 ± 19.2 *	-13 ± 16.5 *	-3.8 ± 5.7 *
**Angle of kyphosis (no. = 24)**	-9.4 ± 25.2	-5.9 ± 22.2	2.3 ± 4.7 *

*, Statistically significant difference vs neutral position; †, statistically significant difference vs 8 kg ASYM. SYM: Symmetrical load; ASYM: asymmetrical load.

Considering the three conditions (8 kg ASYM, 8 and 12 kg SYM), we noticed that the asymmetrical load produced a modification in all the anatomical planes, whereas the symmetrical did not.

The fatigue caused by 'walking with an 8 kg backpack' (Table 5) prompted a further reduction of the angle of lumbar lordosis (18°), a statistically significant reduction of the angle of kyphosis (19°) and a further forward trunk flexion (2 cm). After removing the load, only the angle of lumbar lordosis continued to show a statistically significant difference vs basal conditions.

**Table 5:** Changes from the neutral position pre-fatigue due to the fatigue 7 min test performed.

	**8 kg SYM**	**Without load**
**Trunk rotation (no. = 29)**	0.2 ± 1.5	1 ± 6.5
**Shoulder asymmetry (no. = 29)**	-1.1 ± 5.8	-0.4 ± 6.3
**Frontal plumbline (no. = 29)**	-1.8 ± 7.1	0.9 ± 6.6
**Sagittal plumbline (no. = 24)**	19.5 ± 20.5 *	-1.3 ± 13.3
**Angle of lumbar lordosis (no. = 24)**	-18.4 ± 17.9 *	-2.3 ± 4.7 *
**Angle of kyphosis (no. = 24)**	-19.3 ± 27.7 *	-0.1 ± 4.7

*, statistically significant difference vs neutral position pre-fatigue. SYM: Symmetrical load.

## Discussion

Our results show that loading the spine of a 12-year-old student, symmetrically or otherwise, always prompts postural variations, and they also confirm other findings in the literature [7,14,15,21].

### Effects of symmetrical loads (backpack carried using both straps)

Our findings indicate that forward flexion (which reflects the need to keep the centre of gravity within the support area) is combined with an elongation of the loaded spine (shown by a reduction of lumbar lordosis and kyphosis): it is important to evaluate both these responses, since the elongation could indicate a physiological and correct activation of the paraspinal muscles. No significant differences emerged, in the parameters considered, between the two symmetrical loading conditions (8 and 12 kg), although all the parameter values were increased in the latter. In the 12 kg SYM condition, we also found a significant difference with respect to the basal condition when considering frontal trunk list (4 mm), a difference not emerging in the 8 kg loading condition and which could indicate a difficulty of the postural system in getting the increase of the load adequately over. It is important to recall that what we are looking at here is an instantaneous response that could present variations over longer loading times; having said that, these are habitual loading conditions (SYM) in this population and presumably the response obtained reflects an established adaptation.

We also felt it necessary to consider the construction features of the backpacks carried daily by the schoolchildren: almost all had volume-increasing zips (a factor that could affect load distribution) [8]. Our simulated 'backpack' allowed us to reproduce the 'daily load' in mechanical terms (the forces and couples that are exerted on the subject), but not the 'enlargement effect', which could be a factor in the management of a backpack's contents and therefore in the maintenance of the subject's balance. In our opinion, other studies are needed to investigate 'elongation' and 'forward flexion' responses obtained when using loading systems that reproduce as closely as possible real daily situations.

### Effects of an asymmetrical load (backpack carried using one strap)

In the sagittal plane we observed almost the same responses as those observed in the 8 kg SYM loading condition; the only exception concerned the angle of kyphosis, which was found to be greater than the basal value, a finding that seems to contradict the physiological elongation of the trunk.

In the other planes it was possible to notice an elevation and retropositioning of the loaded shoulder as well as a lateral flexion of the trunk away from the load. This retropositioning could depend on the load itself (too heavy in relation to the functional response capacities of the subjects), or alternatively reflect the need to bring towards the central body line a load that is positioned too laterally. Further studies of the postural effects of asymmetrical loads could relate these parameters to the position of the centre of mass, for example by gathering them with stabilometric data.

### Effects of usual fatigue (walking with an 8 kg backpack worn on both shoulders)

Carrying an 8 kg SYM load accentuates the postural effects in the sagittal plane: after walking (7 minutes), it is possible to observe an increased forward inclination (distance from the plumbline) of the trunk and a further reduction of the angle of lumbar lordosis, as well as a statistically significant reduction of the angle of kyphosis. These changes should be carefully analysed, because they prompt two apparently contradictory responses: i.e., increased forward inclination (distance from the plumbline) and reduced angle of kyphosis. The latter could indicate an increased elongation of the spine but also an awkward (due to fatigue) attempt, when lumbar muscles are no longer able to respond adequately, to achieve a better posture in the sagittal plane (that in turn leads to a further reduction of the angle of lumbar lordosis).

### Postural recovery following removal of the load

The only parameter that does not appear to return to its pre-loading value is the angle of lumbar lordosis. This difference vs baseline, although statistically significant, is not clinically significant as it remains within 2°. Further studies are needed in order to establish whether it warrants more thorough investigation.

### Limitations of the study

This research protocol has been developed to study the postural effects of what has been verified on average in the population studied [6-8]: our studies proved that independently from their body weight, school pupils carry different loads in different school days that drive to an average load quite similar in different classes. Another interesting protocol could have been to compare different loads effects in the same pupils, as it has recently been done [17,21]: we preferred to simulate real conditions. Moreover, many tests would have implied fatigue effects: this is why we chose weights to which the pupils were used, divided in normal (average) and maximal for the usual school week. Anyway, future studies with different protocols should be developed to gather more answers.

Another relevant point is the way the weight has been considered, i.e. as an absolute value and not in percentage of the weight of each student. Again, this point is due to the initial choice of simulating real life situations; the percentage of the weight of the backpack versus body weight created too small groups to give a significant analysis. Another protocol would be for sure able to face this problem. Nevertheless, in real life the backpack weight is related only to school needs and not pupils' weight or choices [6-8].

The backpack simulator is another issue to be considered. Due to the hardware used for evaluation (AUSCAN), we needed to see the spine, and so it was not possible to have a real backpack. We carefully changed individually the length of the straps, so to apply the load in the higher buttocks as usually done by the subjects considered. Obviously, in real life the load applied to the spine can change according to the leverage generated by the distribution of the load in the backpack, as well as to the strap tension [7,8], but this limitation was not avoidable in this study.

### Practical aspects and conclusions

Most of the 12-year-old schoolchildren in a population we previously studied [7] carry their school things in a backpack worn on both shoulders (92%) and the load borne corresponds, on average, to 22% of their body weight [6]; Pascoe et al. [14] found, instead, that their student population preferred the one-strap carrying method (73.2%) and that the average load corresponded to 17.7% of their body weight. It is important to consider the postural response to symmetrical as well as to asymmetrical loads in populations from different backgrounds. Since our aim, in this study, was to explore the postural effects of typical daily loads in a specific population, we considered only two loads: 8 and 12 kg. Our results suggest that a 12 kg load, fairly common in this population (carried at least once a week), seems to push the postural system to its physiological limits. Having said that, the effects of a 12 kg load were studied only in relation to those of an 8 kg load and thus we do not know what findings would emerge considering it in relation to lighter or heavier loads. The asymmetrical load provokes marked variations in posture, and our subjects told us that they found this method of backpack carrying tiring.

The fatigue test (7 minutes' walking) also pushed the postural system to its limits under loading conditions. Hong and Brueggeman [16] conducted a similar evaluation, but they considered loads ranging from 10% to 20% of the subjects' body weight, which do not correspond to the daily values found in our population. Moreover, theirs was a gait analysis and not a postural evaluation; they had their subjects walk for 20 minutes on a treadmill bearing different loads, and concluded, on the basis of changes in biomechanical parameters of gait, that a limit of 10% of body weight should be set. In our study we consistently used an 8 kg load and the walking time was 7 minutes: these values were established on the basis of an *in vivo *evaluation of the schoolchildren's behaviour and therefore reflected more accurately their real everyday situation.

While this paper was under evaluation, Chow and its group produced a considerable amount of work [17-21]. They studied a normal population and an adolescent idiopathic scoliosis one, and progressively evaluated backpack loads of 0, 7.5, 10.0, 12.5 and 15.0% of body weight. Main results with increasing backpack load included: a reduction of walking speed and cadence and an increase in double support time [21]; an increased anterior flexion of the trunk on the pelvis, extension of the head on the trunk, and antero-posterior balance difficulty both in normals and scoliosis patients, even if the last one showed poor balance also in the medio-lateral direction [17]. The authors identified a possible critical load in approximately 10% body weight [21], that presumably should be decreased in scoliosis population [17]. These studies propose results quite similar to ours in terms of antero-posterior difficulty, even if the percentage body weight we studied are much higher due to the everyday reality in our population [6-8]. Moreover, our hardware, that nevertheless required to have a backpacks simulator, allowed a deepening on real spine behaviour, not considering it only as the link between the shoulder and pelvis girdles: this can be considered a limitation as well as an advantage of this study design.

The backpack load effect on schoolchildren posture should be more carefully evaluated in the future. As we already stated in the past following our previous works [6-9], we continue today not to understand why we should have laws who protect workers to carry heavy loads even for a few minutes per day, while we do not look at our children; moreover now, that a recent paper have shown that back pain in youngsters is correlated with back pain in adulthood [5].
